# Optical Characterization of Plasmonic Indium Lattices Fabricated via Electrochemical Deposition

**DOI:** 10.1021/acsaom.2c00188

**Published:** 2023-03-15

**Authors:** Marco Valenti, Merlinde D. Wobben, Yorick Bleiji, Andrea Cordaro, Stefan W. Tabernig, Mark Aarts, Robin D. Buijs, Said Rahimzadeh-Kalaleh Rodriguez, Albert Polman, Esther Alarcón-Lladó

**Affiliations:** † Center for Nanophotonics, NWO-Institute AMOLF, Science Park 104, 1098 XG Amsterdam, The Netherlands; ‡ Institute of Physics, University of Amsterdam, Science Park 904, 1098 XH Amsterdam, The Netherlands

**Keywords:** plasmonic, indium, metal nanoparticle array, electrochemical deposition, surface lattice resonance (SLR), UV-vis plasmonics

## Abstract

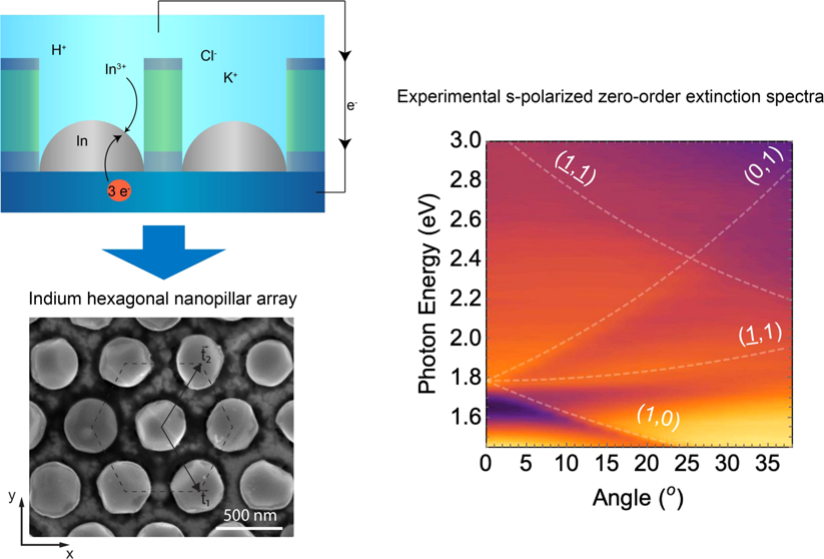

The optical properties of periodic metallic nanoparticle lattices have found many exciting applications. Indium is an emerging plasmonic material that offers to extend the plasmonic applications given by gold and silver from the visible to the ultraviolet spectral range, with applications in imaging, sensing, and lasing. Due to the high vapor pressure/low melting temperature of indium, nanofabrication of ordered metallic nanoparticles is nontrivial. In this work, we show the potential of selective area electrochemical deposition to generate large-area lattices of In pillars for plasmonic applications. We study the optical response of the In lattices by means of angle-dependent extinction measurements demonstrating strong plasmonic surface lattice resonances and a good agreement with numerical simulations. The results open avenues toward high-quality lattices of plasmonic indium nanoparticles and can be extended to other promising plasmonic materials that can be electrochemically grown.

## Introduction

Nobel-metal nanostructures are key building blocks for the manipulation of light at small length scales and have found many applications in the fields of imaging,^1^ nanoscale lasing,^2^ sensing,^3^ surface-enhanced Raman scattering,^4^ and enhanced photo(electro)chemical^5−7^ and photovoltaic devices.^8,9^ When optically excited, metal nanoparticles can concentrate light into nanoscale near fields with light intensity enhancements as high as 10^3^ owing to the creation of localized surface plasmon resonances (LSPRs).^10^ Strong light scattering as well as Ohmic dissipation in the metal result in plasmon lifetimes of tens of femtoseconds, corresponding to spectral linewidths of typically 50–100 nm.^11^ When nanoparticles are arranged in periodic arrays, diffracted orders radiating in the periodicity plane (Rayleigh anomalies)^12−15^ can couple to localized surface plasmons and form surface lattice resonances (SLR).^16^ SLRs, in turn, can have very small linewidths of a few nanometers, opening exciting opportunities for enhanced biochemical imaging and sensing.^13^ For the most commonly used plasmonic metals Ag and Au, the particle and array resonance frequencies typically lie in the visible and near-infrared spectral range and are tunable by varying nanoparticle size and shape, and interparticle distance.^17,18^

A key spectral range that is not well covered by plasmonic metals is the ultraviolet. Yet, many applications in sensing, imaging, (photo-)catalysis, and more would strongly benefit from the use of highly concentrated optical near fields in the UV. Indium is a plasmonic metal with optical constants that enable the creation of plasmon resonances from the visible to the ultraviolet.^19−21^ Compared to other UV-plasmonic metals, like Al or Tl, In offers an extended range of wavelengths with intense near fields from the near-UV to the visible.^21,22^ Contrary to the case for Ag and Au, no wet-chemical colloidal synthesis processes to fabricate indium colloids are known. Furthermore, the controlled growth of indium nanostructures with vapor-based methods, as often used for other plasmonic metals, is difficult owing to its high vapor pressure and low melting point. Earlier studies have shown that arrays of indium nanopillars can be grown electrochemically from aqueous solutions at room temperature on a templated substrate.^23^ However, so far little is known about the optical properties of these arrays for plasmonic applications. Indeed, it is well known that the uniformity of electrochemical crystal growth in confined geometries is limited due to the strong three-dimensional variations of electric fields and transient diffusion profiles in nanotemplates,^23^ which in turn, is expected to have a strong effect on the plasmonic properties.

Here, we combine substrate conformal soft-imprint lithography with electrochemical growth to realize large-area indium nanoparticle arrays with well-defined dimensions. We find that our templated growth process creates well-defined hexagonal array of particles with a mean particle height of 235 nm with a standard deviation of 60 nm. To prove the applicability of the proposed fabrication strategy to plasmonic devices, we used angle-dependent extinction measurements to demonstrate the strong plasmonic response of the indium nanoparticle arrays due to coupling to plasmonic surface lattice resonances (SLRs). We show using finite difference time domain (FDTD) simulations that the measured spectral features agree well with the theoretical spectrum. Our results open exciting new opportunities to grow indium nanoparticle arrays with well-defined optical resonances that cover a broad ultraviolet–visible spectral band.

## Results and Discussion

**Figure 1:**
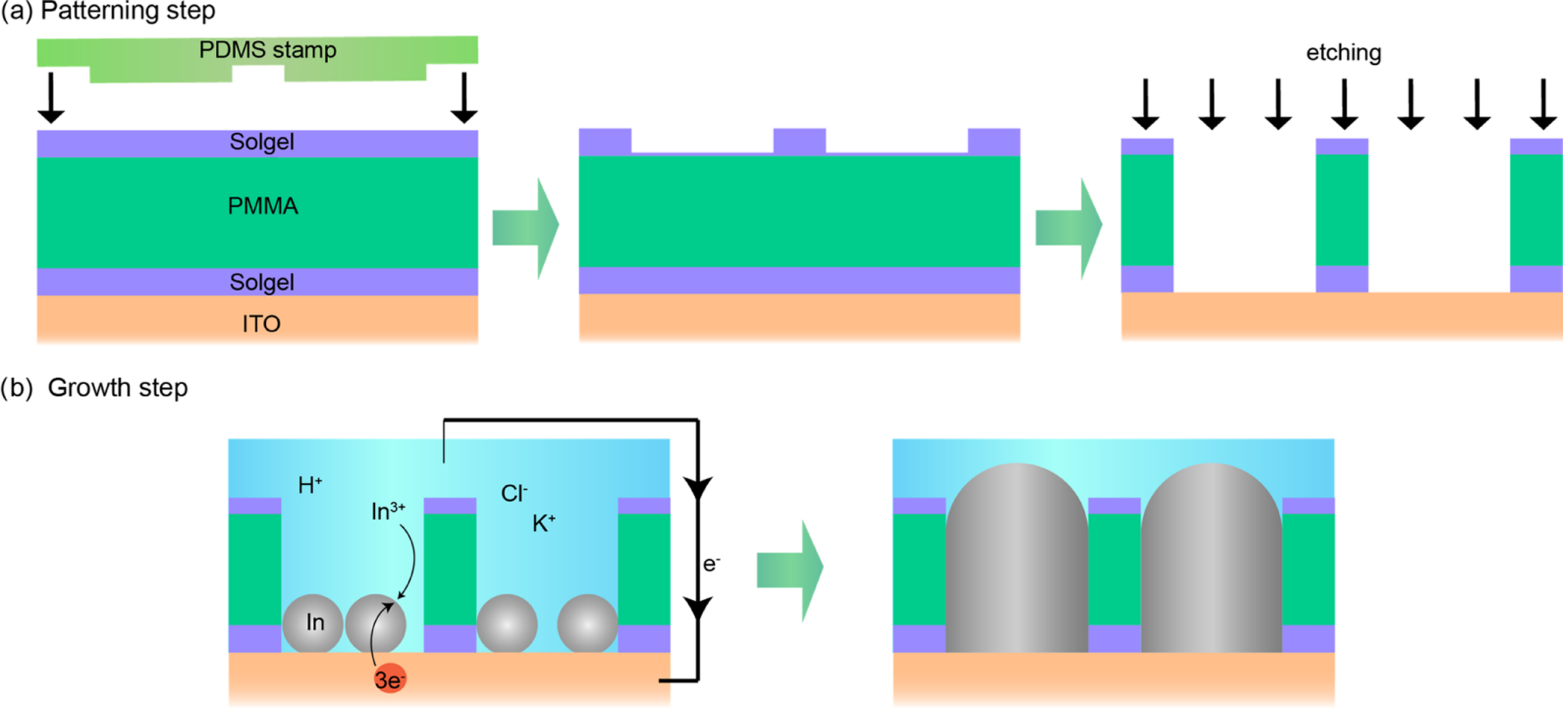
Fabrication steps for the electrochemical growth of the indium pillar hexagonal lattices. (a) A SiO_2_/PMMA/SiO_2_ multilayer is deposited on an ITO/glass substrate and the SiO_2_ sol–gel top layer is patterned using SCIL to create a two-dimensional (2D) array of holes in the top film. (b) The imprinted substrate is used as a working electrode for indium electrodeposition. The growth occurs in an In-ion-containing electrolyte at the ITO-exposed areas under cathodic conditions (−1.3 V vs Ag/AgCl) for 33.7 s (see the Methods section for more details).

The In pillar arrays were fabricated using selective area electrochemical deposition. In this technique, an insulating mask of SiO_2_/PMMA/SiO_2_ is made on indium tin oxide (ITO)-coated glass using substrate conformal soft-imprint lithography (SCIL) (Figure 1a). SCIL is a scalable nanofabrication method that creates high-fidelity nanostructures over up to 6″ wafers and can create nanoscale features with characteristic dimensions as small as 6 nm.^24^ Upon imprinting, three etching steps create an array of cylindrical holes in the SiO_2_/PMMA/SiO_2_ mask on top of the ITO substrates.

The nominal hole radius is 170 nm, and the hexagonal array has a pitch of 534 nm, which is designed to generate in-plane Rayleigh anomalies in the UV–visible spectral range (see Dispersion section in the Supporting Information). Subsequently, the patterned substrate is exposed to an aqueous electrolyte containing In^3+^ ions in a custom-made electrochemical cell (Figure 1b). Indium electrodeposition is restricted to the electrolyte-exposed ITO surface upon application of a constant potential in a three-electrode configuration for about 30 s (see the Methods for detailed information on the electrochemical deposition). After nucleation and coalescence, a single particle per hole emerges, establishing a vertical growth that is restricted by the hole dimensions. Finally, the PMMA and top SiO_2_ mask is removed by sonicating the sample in acetone at 30 °C for 30 min.

**Figure 2:**
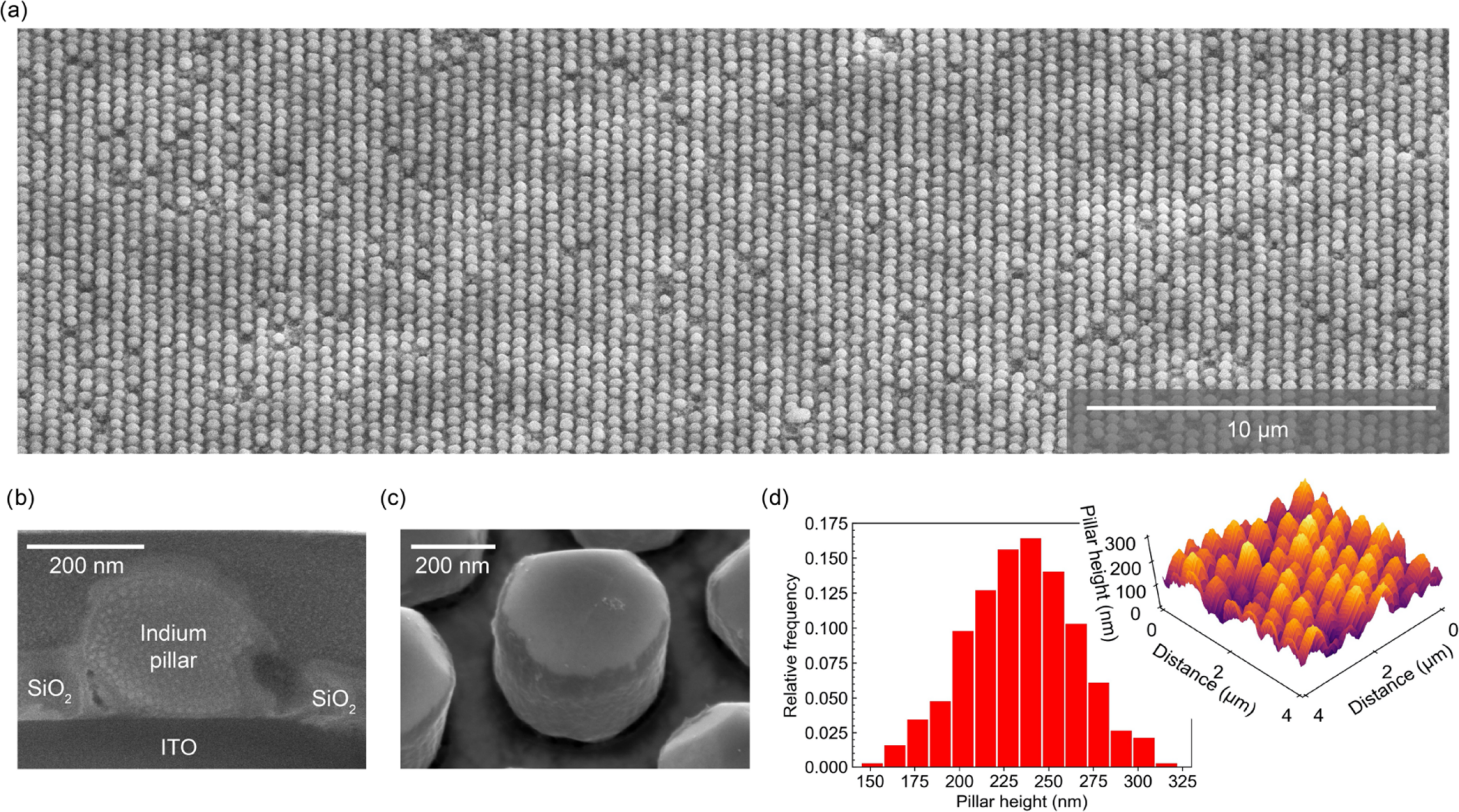
Morphological characterization of the electrochemically grown indium pillar hexagonal lattice. (a) Large-area SEM overview at a 30° tilt. (b) Cross-sectional SEM image taken at a 52° tilt of a single pillar. The cross section was fabricated with focused ion beam milling, where the sample surface was locally protected by a Pt layer. (c) Close-up SEM image of a single indium pillar taken at a 35° tilt after mask removal. (d) Atomic force microscopy (AFM) topography (inset) and pillar height histogram of a representative area in the sample.

A scanning electron microscopy (SEM) image of the resulting periodic In nanoparticle array is shown in Figure 2a. The nanoparticles fill the pattern with a high yield (95.5%), where the partial or absent In deposition is considered as defects (see the Supporting Information). Figure 2b shows the cross section of an indium pillar after the removal of the top sol–gel and PMMA layers. While the hole does not appear to be uniformly filled, we cannot rule out ion beam damage during the focused ion beam (FIB) milling. Indeed, the tilted SEM image of a pillar after mask removal in Figure 2c reveals straight vertical walls, which indicates that the shape of the mask is well transferred to the electrochemically grown nanopillar. This, in turn, creates new avenues to tailor the shape of the single indium nanoparticle for further plasmonic resonance engineering.

The pillar height distribution is measured with atomic force microscopy (AFM). A representative topography map is shown in the inset of Figure 2d. Empty holes are used as the reference of zero height. Figure 2d shows the histogram for the pillar height measured in an AFM scan area of 10 × 10 μm^2^, revealing a normal distribution of pillar height centered at about 235 nm and a standard deviation of 60 nm. Such inhomogeneous particle height or polydispersity is not unusual in dense selective area electrochemical deposition and has been explained by interparticle competition for diffusing ions.^23^ More specifically, if the particle nucleation is not simultaneous and homogeneous, the pillars grown from the first nuclei will consume the ions of their neighbors, inhibiting or further delaying the growth of those. Both the height polydispersity and the dome-like top (i.e., the highest point at the center of the pillar) obtained here indicate interparticle competition for diffusing ions.

**Figure 3:**
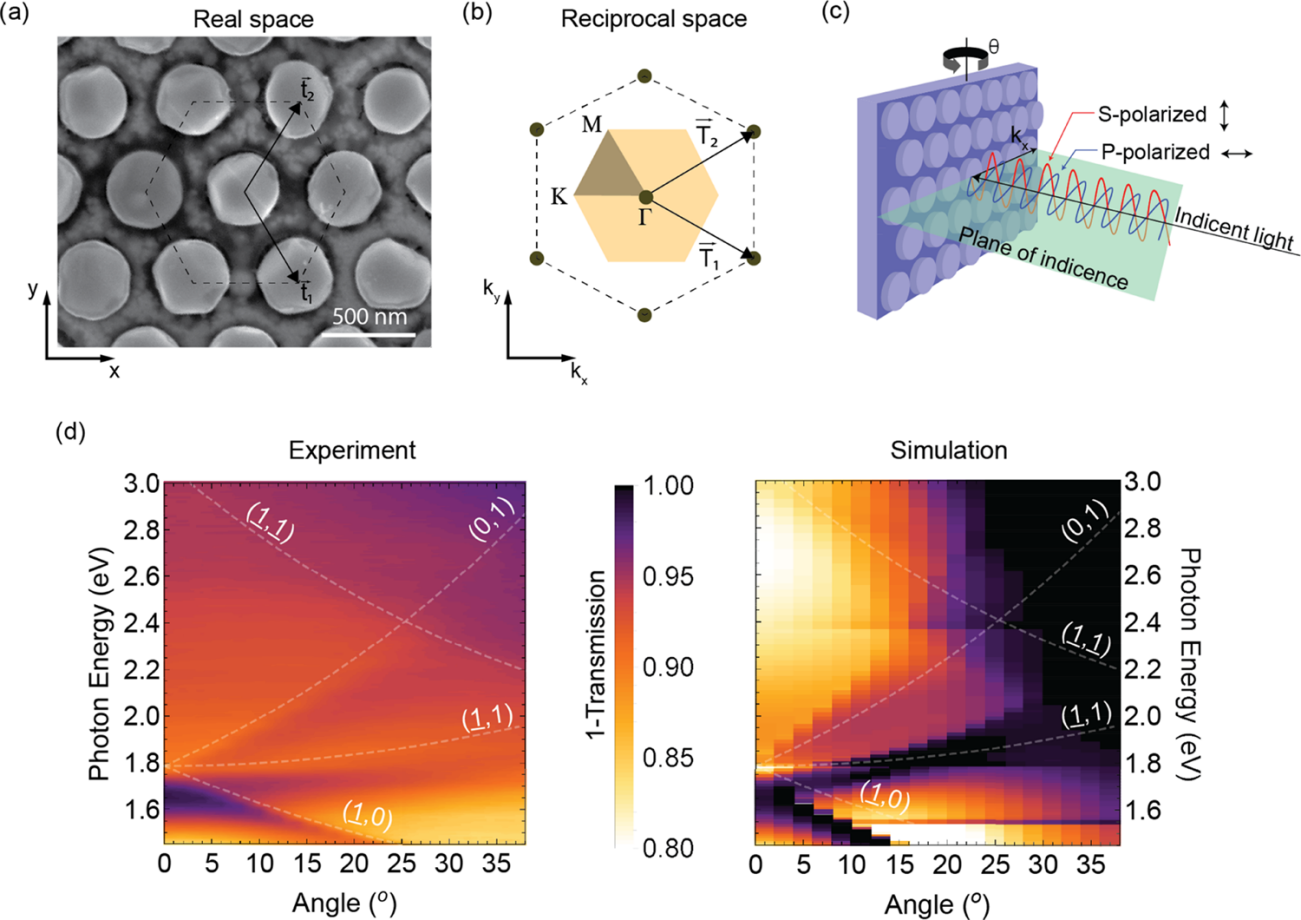
Angle-dependent transmission of the hexagonal indium nanopillar array. (a) SEM image of the hexagonal nanopillar array oriented according to the laboratory coordinates and with indicated direct lattice vectors *t*_1_ and *t*_2_. (b) Corresponding reciprocal space representation of the hexagonal array along with the Brillouin zone (shaded area), characteristic high-symmetry points (Γ, *M*, and *K*), and lab reciprocal coordinates (*k_x_* and *k_y_*). (c) Schematic of the measurement configuration. By rotating the sample as indicated by the black arrow, *k_x_* is increased. In our experiments, we use the s-polarization configuration. (d) Measured and calculated *s*-polarized zero-order extinction spectra as a function of angle θ. The dashed lines indicate the dispersion for the different diffraction orders, indicated by labels. The straight lines at 1.55 and 2.4 eV are simulation artifacts.

To study the optical properties of the as-grown indium nanoparticle lattice and to assess its potential for plasmonic applications, the optical transmittance (*T*) spectra of the zeroth-order diffraction are measured as a function of the incident angle. These measurements were carried out under *s*-polarization conditions with the sample embedded in a refractive index (RI) matching oil to reduce substrate-to-superstrate index contrast that is known to suppress long-lived lattice resonances.^25^Figure 3a–c shows the array’s direct lattice, reciprocal lattice, and experimental configuration, respectively. In the experiment, we rotate the sample around the y-axis by an angle θ, which introduces an in-plane wave vector . Figure 3d shows the measured and simulated extinction (1 – *T*), respectively, based on the zero-order transmitted light as a function of incidence angle to the indium pillar array. Measurement series done on different days did not show any sign of sample degradation, indicating the robustness of the In material. For the simulation, we have used an FDTD solver^26^ to obtain the zero-order transmission of a hexagonal array (pitch of 534 nm) of indium pillars of 170 nm in diameter, 225 nm in height, and a rounded top surface, based on the nominal pattern parameters and measured pillar geometry. We expect that the surface of the In pillars is covered by a <4 nm thick native oxide layer, which we have not taken into account for the simulations. For simplicity, we also consider the array to be suspended in a dielectric environment of refractive index *n* = 1.5, thus neglecting the ITO. See the Methods section and the Supporting Information file for more details, including the effect of ITO thin layer on the transmission.

We observe a clear extinction peak in the experimental angle-dependent extinction spectra (dark region in Figure 3d around 1.6 eV at normal incidence) splitting into three extinction peaks with increasing angle. The dispersion of the three extinction peaks matches well with that predicted for the Rayleigh anomalies (RA) related to the (−1, 0), (−1, 1), and (0, 1) diffraction orders, as indicated by the dashed gray curves. In fact, these three diffraction orders are strongly scattered in-plane, giving rise to strong plasmonic surface lattice resonances (SLRs). This, in turn, implies a prolonged interaction between light and the lossy In nanoantennas resulting in enhanced absorption and hence a peak in extinction. The observed redshifted dip in extinction with respect to the RAs suggests of the coupling with localized plasmon resonances associated with the In nanopillars (LSPR). The RA dispersion has been calculated by considering the configuration indicated in Figure 3b,c and taking into account the surrounding medium (see more details in the Supporting Information).

It is worth mentioning that the observed resonances in Figure 3d are weaker than those typically reported for highly homogeneous plasmonic lattices.^13,27^ The low-quality factor of the resonances is an indication of short-lived SLRs, likely arising from the height polydispersity of the array. It has been shown that SLRs can be significantly broadened or do even not show up in particle arrays with large variations in particle size.^28^ Indeed, the simulated extinction of a uniform array shows very similar spectral features as in the experiment but with a stronger contrast, indicating a much higher resonance quality factor.

**Figure 4:**
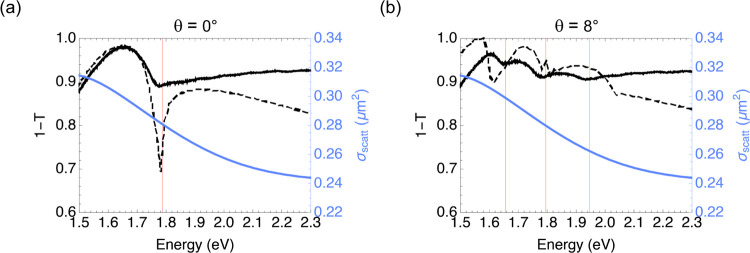
Measured and simulated extinction spectra. (a) Measured (solid black) and simulated (dashed black) s-polarized zero-order extinction spectra at normal incidence. The solid blue line shows the scattering cross-section spectrum of a single indium nanoparticle. The solid red line indicates the onset of diffraction (degenerate orders (−1, 0), (−1, 1), and (0, 1)). (b) Same comparison as in (a) with an s-polarized light impinging on the sample at θ = 8°.

To compare the experimental data and its simulated counterpart in more detail, we examined crosscuts taken from the color maps in Figure 3d at normal incidence and at θ = 8°. Figure 4 shows such a comparison demonstrating a good match between experiment and simulation for both angles of incidence. The spectral position of the RAs in the measured extinction compares well with what is expected from the lattice geometry. On the other hand, the resonance linewidths are much broader due to the dielectric asymmetry given by ITO (see the SI for more details) and the described lattice inhomogeneities. These inhomogeneities result in radiative damping, which broadens the resonance linewidth as observed in Figure 4a.

Interestingly, the extinction dips related to the SLR display an asymmetrical line shape typical of Fano-type resonances. This is due to the interference of the broad resonant behavior of the single In nanoparticle (blue solid lines in Figure 4a,b) with the sharper plasmonic surface lattice resonances induced by the grating periodicity.

While still limited by size dispersity, the measurements show a clear plasmonic lattice response and thus demonstrate the potential of the proposed fabrication scheme for future high-quality and large-scale indium-based plasmonic devices in the UV-vis wavelength range.

## Conclusions

We have presented a novel electrochemical strategy to fabricate large-area indium nanoparticle arrays, having a well-controlled size. These In nanoparticle arrays exhibit a strong interaction between the individual particle and the lattice plasmonic resonances. We find that ∼95% of the holes were filled with indium pillars as constrained by the mask, having an average height of 235 nm with a standard deviation of 60 nm.

To assess the potential of electrochemical grown indium nanoparticle lattices for plasmonics, we perform angle-dependent transmittance measurements using *s*-polarized light and using an RI-matching oil. The entire spectrum is dominated by SLRs with angle-dependent features containing more structural and material information. We have used the morphology from SEM and the electrical permittivity of pure indium to simulate the optical response of single-height indium pillar lattices. We have compared these monodispersed simulated transmittance spectra to the experimentally obtained spectra for two incident angles of 0 and 8°, and we have shown a good agreement of the measured spectra with the FDTD simulated ones of single-height indium pillar arrays despite the polydispersity and defects.

Our results demonstrate the possibility of realizing ordered lattices of indium plasmonic nanoparticles with controlled individual size and shape. Given the low-cost facile fabrication, this work opens new avenues in the design and fabrication of large-area plasmonic metasurfaces that operate from the UV to the visble. Future extensions of this work may leverage the indium mask-constrained growth to tailor even further the shape of the nanoparticle and hence give a powerful knob to tailor plasmonic resonances.

## Methods

### Fabrication

An SCIL stamp is pressed onto the sample (75 nm sol–gel layer/300 nm PMMA/32 nm sol–gel/ITO/glass), where only the topmost layer is imprinted. To expose the ITO, a short CHF_3_ + Ar etching is performed, followed by PMMA etching with O_2_ and subsequent CHF_3_ + Ar etching to remove the bottom SiO_2_ layer. The imprinted substrate is used as a working electrode for In electrodeposition in a 2 mL three-electrode cell with a Pt wire counter electrode and a Ag/AgCl (leakless miniature ET062, EDAQ, 0.197 V vs SHE) reference electrode. The growth occurs in the ITO-exposed holes of the substrate under cathodic conditions from an aqueous solution of 0.05 M InCl_3_, 0.2 M KCl, and 0.005 M HCl (pH 2.5).^23^ A potential of −1.3V vs Ag/AgCl is applied for 33.7 s (see Figure 2 in the Supporting Information). After nucleation and coalescence, a single particle per hole emerges and periodicity is established. The pillars grow vertically following the trench.

## Data Availability

The data that support the findings of this study are available from the corresponding author upon reasonable request. All codes produced during this research are available from the corresponding author upon reasonable request.

## Supporting Information Available

Additional experimental and theoretical details and methods (PDF)
