# Supplementary material for: Optical Characterization of Plasmonic Indium Lattices Fabricated via Electrochemical Deposition
Source: ACS Appl Opt Mater. 2023 Mar 15;1(3):753–8. doi: 10.1021/acsaom.2c00188 (PMC10043931; doi:10.1021/acsaom.2c00188)
Supplement: Supplementary file 1 — ot2c00188_si_001.pdf [file ot2c00188_si_001.pdf]

Supporting information: Optical characterisation of plasmonic indium lattices fabricated via electrochemical deposition

Marco Valenti,[†,‡] Merlinde D. Wobben,[†,‡] Yorick Bleiji,[†] Andrea Cordaro,[†] Stefan Tabernig,[†] Mark Aarts,[†] Robin D. Buijs,[†] Said Rahimzadeh-Kalaleh Rodriguez,[†] Albert Polman,[†] and Esther Alarcon Llado[*,†]

†*Center for Nanophotonics, NWO-Institute AMOLF, Science Park 104, 1098XG, Amsterdam, The Netherlands*

‡*Contributed equally to this work*

E-mail: e.alarconllado@amolf.nl

Rayleigh anomaly dispersion curves

The RA dispersion curve calculations are based on the geometry of the array which has a nearest neighbour separation Λ of 534 nm as shown in the main text. The direct and reciprocal lattice vectors as shown in the main text are described as:

$$\mathbf{t_1} = \frac{\Lambda}{2}\hat{x} - \frac{\Lambda\sqrt{3}}{2}\hat{y} \tag{1}$$

$$\mathbf{t_2} = \frac{\Lambda}{2}\hat{x} + \frac{\Lambda\sqrt{3}}{2}\hat{y} \tag{2}$$

$$\mathbf{T_1} = \frac{2\pi}{\Lambda}\hat{k}_x - \frac{2\pi}{\Lambda\sqrt{3}}\hat{k}_y \tag{3}$$

$$\mathbf{T_2} = \frac{2\pi}{\Lambda}\hat{k}_x + \frac{2\pi}{\Lambda\sqrt{3}}\hat{k}_y \tag{4}$$

The sample was rotated around the y-axis from θ 0 to 46° in steps of 2°. The RA energies are solutions to

$$E = \frac{\hbar c}{n}|\mathbf{k}_{//} + (m_1\mathbf{T_1} + m_2\mathbf{T_2})|, \tag{5}$$

where m_1 and m_2 are the diffraction orders, $k_{//} = \frac{E}{\hbar c} \cdot \sin\theta\hat{k}_x$ and $n = 1.5$ is the refractive index of the surrounding medium.

Fabrication

Soft conformal imprint lithography

The In plasmonic lattices were fabricated by Substrate Conformal Imprinting Lithography (SCIL) as follows: ITO on glass substrates was cleaned with soap and subsequent sonication in acetone and IPA. A layer of silica sol-gel (SCIL Nanoimprint solutions T1100) was spin-coated (30-40 nm) onto the ITO substrate. This was followed by curing at 150°C for 1 min. Next, a layer of PMMA (PMMA 950k A8 in anisole, 250 nm) was spin-coated and baked

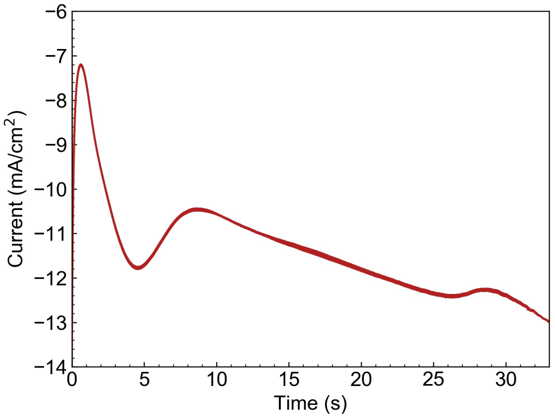

Figure 1: Chronoamperometry plot of electrodeposition in hexagonal imprinted substrate.

at 150°C for 2 min. A brief and weak O_2–plasma was performed before another layer of silica sol-gel was spin-coated (75 nm) and imprinted with a triple-layer PDMS stamp. The stamp was carefully removed after a curing time (room temperature) of 6 minutes. The pattern was transferred into the PMMA by a 3-step reactive-ion-etching (RIE) process: first a fluorine-based (CHF_3 + Ar) breakthrough etch that removed the thin residual sol-gel layer that remained under the recessed features after the imprint. Then, an O_2-based plasma etch was performed to pattern the PMMA using the sol-gel as a hard mask. Finally, another fluorine-etch removed the final sol-gel layer.

Electrochemical deposition

The imprinted substrate is used as a working electrode for In electrodeposition in a 2 mL three-electrode cell with a Pt wire counter electrode and an Ag/AgCl (0.21 V vs SHE) reference electrode. The deposition area was $0.059cm^2$, limited by our electrochemical cell aperture. The growth occurs in the ITO-exposed holes of the substrate under cathodic conditions from an aqueous solution of 0.05 M InCl3, 0.2 M KCl, and 0.005 M HCl (pH 2.5)20. A potential of -1.3 V vs ref. is applied for 33.7 s. Figure 1 shows the electrochemical current during the growth time. During the growth, the solution was not stirred. After nucleation and coalescence, a single particle per hole emerges and periodicity is established.

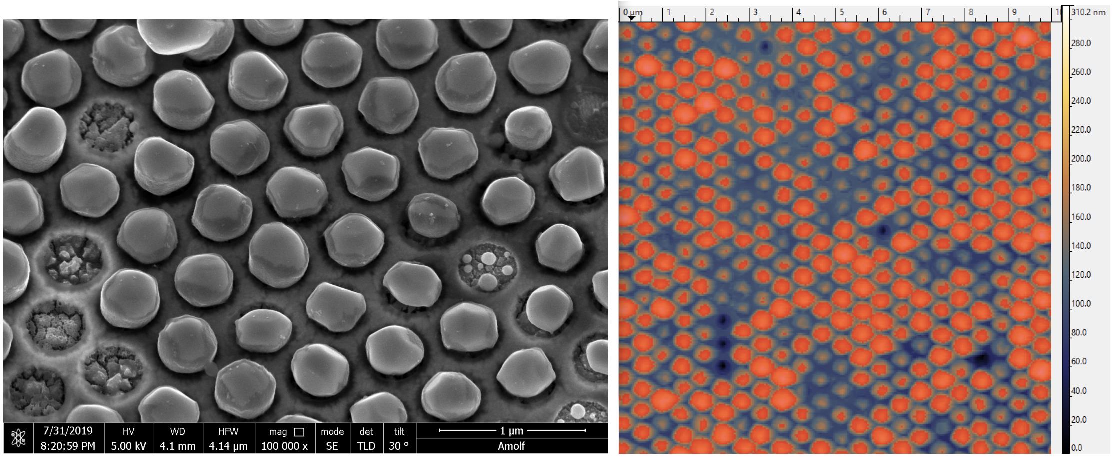

Figure 2: (left) SEM image of the lattice showing defects.(right) AFM map with empty holes from which the baseline at the ITO surface was extracted.

The pillars grow vertically following the trench. In the exposed area, some regions presented defects, indicated by the lack of pillar growth (Figure 2). Defects are used as reference for the height measurements with the AFM.

FDTD simulations

To simulate the reflection and transmission spectra, Maxwell's Equations were solved numerically, using an FDTD-code (Ansys Lumerical - FDTD: 3D Electromagnetic Simulator).[1] The optical properties for In was taken from[2] and approximated by fitting curves to the datasets.[1]

The shape of the In pillars was approximated by using a rounded cylinder object, with only the upper dome of 50 nm in radius. The pillar radius and total height were 170 nm and 225 nm, respectively. The hexagonal array was achieved by considering a rectangular simulation area, as indicated in Figure 4.

Periodic boundary conditions were used for the in-plane boundaries, that were overwritten by the BFAST plane wave source under off-normal incidence conditions. In all cases, antisymmetric boundary conditions were kept for the boundaries parallel to the magnetic field (y_{max} and y_{min}). Perfectly matched layers (PMLs) are used at the boundaries along the source injection plane. Two frequency domain field and power-monitors were used to

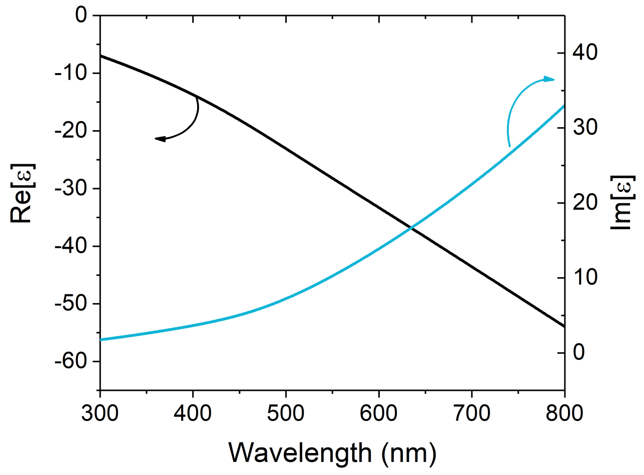

Figure 3: Optical constants of Indium used in the simulations in this work.[2]

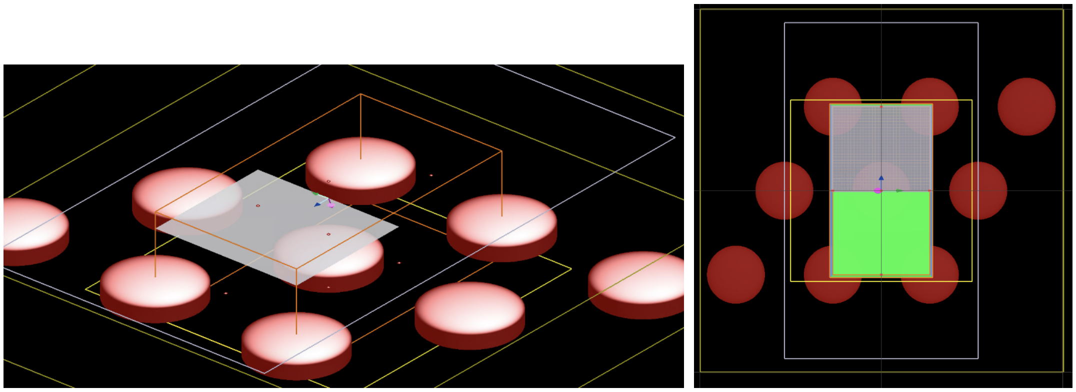

Figure 4: Screenshots of the FDTD simulation geometry (tilted and top views, from left to right).

obtain reflected and transmitted spectra. The mesh refinement was set to conformal variant 1 to account for the metals in the simulations, and the mesh size was determined by mesh convergence testing with a minimum mesh step of 1 nm.

The transmittance was obtained by a grating transmission monitor. The script below has been used to extract the total and zeroth-order transmission:

```
mname="transmission"; # monitor name
mmname="reflection";
nvar=0; #n-order
mvar=0; #m-order
```

```
f=getdata(mname,"f"); # get frequency vector
T=transmission(mname); # get total transmission
Gnm = matrix(length(f)); # initialize matrix
R=transmission(mmname);
#get 0,0 grating order for each frequency
for (i=1:length(T)) {
  N=gratingn(mname,i); # grating order numbers
  M=gratingm(mname,i);
  temp=grating(mname,i);
  Gnm(i) = temp(find(N,nvar),find(M,mvar)); # select 0,0 grating order
}
lambdaValues=c/f*1e9;
Ttot=-T;
Tnm=-Gnm*T;
plot(lambdaValues,Ttot,Tnm,"wavelength (nm)","power");
legend("total transmission","n="+num2str(nvar)+",m="+num2str(mvar)+" grating
    transmission");
exportFilename="h230_"+"T"+num2str(nvar)+num2str(mvar);
matlabsave(exportFilename,lambdaValues,Ttot,Tnm,R,nvar,mvar);
# combine both vectors into a single 2D matrix to be output to file
data_to_print=[lambdaValues,Ttot,Tnm,R];
# write the data to the file
write("filename.txt",num2str(data_to_print));
```

ITO effects on the transmission

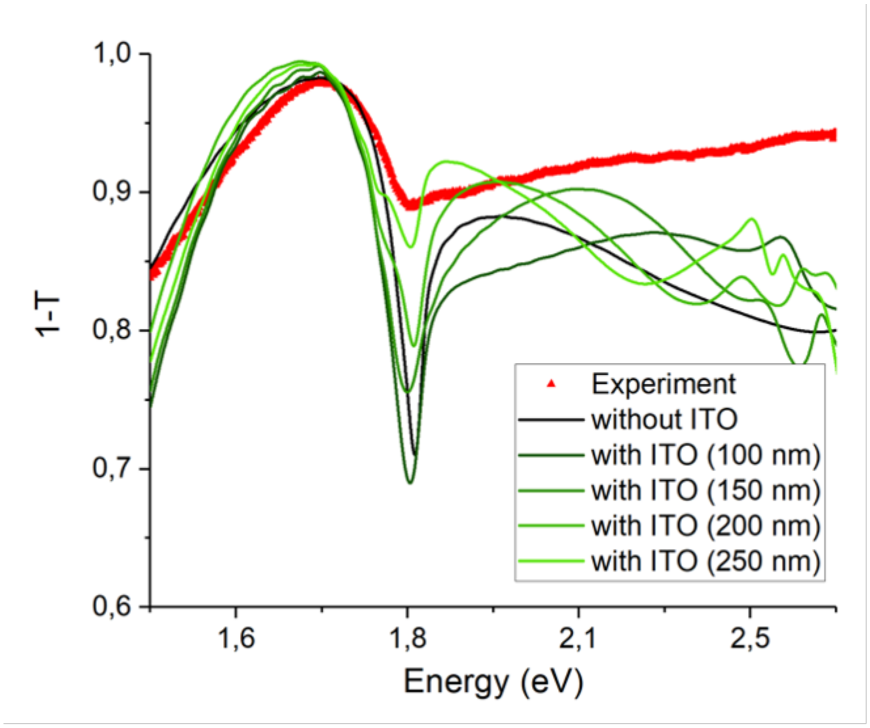

Figure 5: Extinction at normal incidence for the In array. Measured in red, and calculated is in shades of green, depending on the ITO thickness (indicated by the legend). The thicker the ITO, the less pronounced is the extinction peak at 1.8 eV.

Optical characterisation

To experimentally determine the plasmonic lattice modes dispersion, angle-resolved transmission was measured. The data was collected with a Spectra Pro 2300i spectrometer equipped with a Pixis 400 CCD. The sample was mounted on a rotating stage and illuminated with polarized collimated white light from a SuperK EXTREME/FIANIUM supercontinuum laser. The transmitted light was collected by an integrating sphere and sent to the spectrometer through a multimode fiber. The zeroth–order transmission was selected using an iris at the entrance of the integrating sphere.

To ensure a symmetric dielectric environment, the sample was immersed in a Nikon immersion oil (n=1.518). Figure 6 shows the effect on transmission of using the oil compared

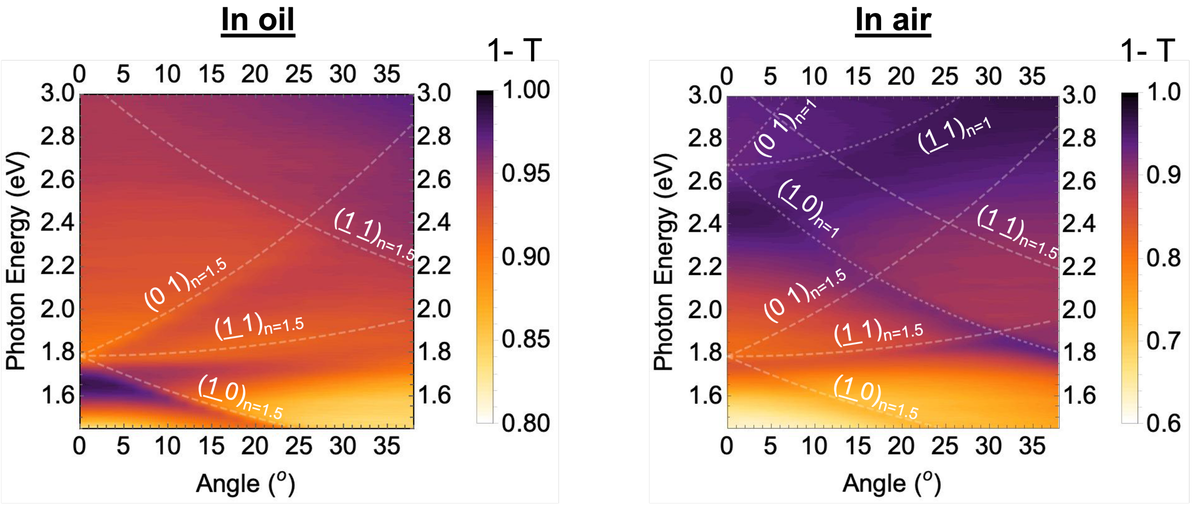

Figure 6: Extinction as a function of angle when the sample is in oil and in air.

to the case where the sample is in air. In the latter, diffraction modes are observed for both glass and air, which is in detriment to the surface lattice resonance quality.

References

(1) Lumerical Inc., FDTD: 3D Electromagnetic Simulator. https://www.lumerical.com/.

(2) Koyama, R. Y.; Smith, N. V.; Spicer, W. E. Optical Properties of Indium. *Phys. Rev. B* **1973**, *8*, 2426–2432.
